# Strategies for the preparation of non-amplified and amplified genomic dengue gene samples for electrochemical DNA biosensing applications

**DOI:** 10.1039/d1ra06753b

**Published:** 2021-12-20

**Authors:** Jahwarhar Izuan Abdul Rashid, Nor Azah Yusof, Jaafar Abdullah, Rafidah Hanim Shomiad @ Shueb

**Affiliations:** Department of Chemistry and Biology, Centre for Defence Foundation Studies, National Defence University of Malaysia Sungai Besi Camp 57000 Kuala Lumpur Malaysia jahwarhar@upnm.edu.my; Department of Chemistry, Faculty of Science, Universiti Putra Malaysia Serdang Selangor 43400 Malaysia; Institute for Research in Molecular Medicine (INFORMM), Universiti Sains Malaysia 16150 Kubang Kerian Kelantan Malaysia

## Abstract

The application of electrochemical DNA biosensors in real genomic sample detection is challenging due to the existence of complex structures and low genomic concentrations, resulting in inconsistent and low current signals. This work highlights strategies for the treatment of non-amplified and amplified genomic dengue virus gene samples based on real samples before they can be used directly in our DNA electrochemical sensing system, using methylene blue (MB) as a redox indicator. The main steps in this study for preparing non-amplified cDNA were cDNA conversion, heat denaturation, and sonication. To prepare amplified cDNA dengue virus genomic samples using an RT-PCR approach, we optimized a few parameters, such as the annealing temperature, sonication time, and reverse to forward (R/F) primer concentration ratio. We discovered that the generated methylene blue (MB) signals during the electrochemical sensing of non-amplified and amplified samples differ due to the different MB binding affinities based on the sequence length and base composition. The findings show that our developed electrochemical DNA biosensor successfully discriminates MB current signals in the presence and absence of the target genomic dengue virus, indicating that both samples were successfully treated. This work also provides interesting information about the critical factors in the preparation of genomic gene samples for developing miniaturized PCR-based electrochemical sensing applications in the future. We also discuss the limitations and provide suggestions related to using redox-indicator-based electrochemical biosensors to detect real genomic nucleic acid genes.

## Abstract 2

The application of electrochemical DNA biosensors in real genomic sample detection is challenging due to the existence of complex structures and low genomic concentrations, resulting in inconsistent and low current signals.

## Graphical abstract

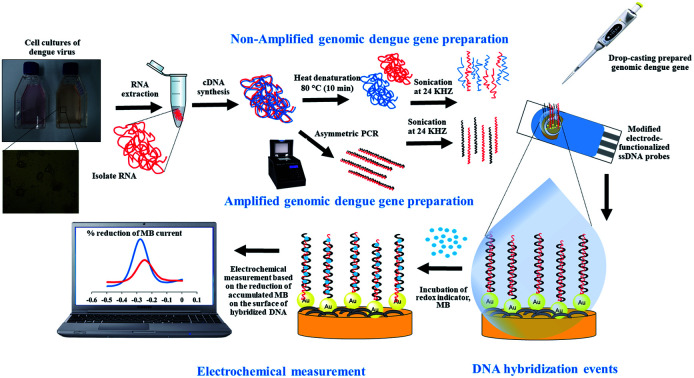

## 1. Introduction

Dengue rapid diagnostic tests (RDTs) are becoming highly attractive as they offer the fast diagnosis of the dengue virus due to their ease of use and inexpensiveness. While various commercial dengue RDTs based on the detection of IgM, IgG, or dengue virus non-structural protein 1 (NS1) are available on the market, some drawbacks associated with sensitivity and selectivity mean that laboratory-based RT-PCR procedures are still required for confirmatory testing. As reported by Rashid *et al.*,^1^ it has been stated that some commercial RDTs exhibit poor sensitivity during NS1 and IgM detection, ranging from 37% to 68% and from 20.5% to 77.8%, respectively. Although some commercial IgG/IgM and NSI-based dengue RDTs can show enhanced sensitivity, they need multistep sample preparation processes. Furthermore, the RDT results are interpreted based on the presence of two individual test lines that can only be used to provide a yes/no answer and do not determine the severity or stage of a dengue virus infection.

Due to all these circumstances, researchers have recently focused on the development of point-of-care quantitative biosensors for dengue virus diagnosis with high selectivity and specificity, portability, and ease of use.^2–5^ In terms of sensor biorecognition elements, nucleic acid or DNA biosensors are preferred over enzyme-, antibody-, or microorganism-based biosensors due to their specific binding through DNA hybridization events, high stability, ease of synthesis, minimal batch-to-batch variation, and biocompatibility.^6^ The underlying principle behind the mechanism of a DNA biosensor is based on the detection of a DNA hybridization event, which is the process of joining two single-stranded DNA (ssDNA) strands between immobilized ssDNA probes with the formation of target double-stranded complementary DNA (dsDNA). The changes in properties of ssDNA and dsDNA before and after DNA hybridization are detectable using different transducer platforms, such as electrochemical, optical,^7^ or piezoelectric^8^ platforms. Electrochemical detection has shown great potential for use with DNA biosensors in point-of-care (POC) dengue devices because of the portability, ease of operation, cost-effectiveness, quantitative analysis, and possibility of miniaturization.^9^

In the past few years, our group has focused on electrode modification using nanomaterials such as silicon nanowires (SiNWs) and gold nanoparticles (AuNPs) to create novel DNA immobilization matrices for electrochemical DNA sensing.^10,11^ Because of the limitations of label-free electrochemical detection in terms of sensitivity,^12^ we have employed a redox DNA hybridization indicator to monitor the current signals from DNA hybridization events. Various redox DNA indicators that are available include ethidium bromide^13^ (EtBr), Hoechst 33258,^14^ methylene blue, acridine orange,^15^ RuHex^3+^,^16^ ferrocene,^17^ daunomycin,^18^ Meldola blue,^19^ and Co(phen)_3_^3+^,^20^ and others have also been documented. Erdem and colleagues^21^ reported the first work on the high discrimination potential of MB binding properties, and they found out that MB demonstrated higher affinity toward guanine bases in ssDNA (before DNA hybridization) compared to dsDNA (after DNA hybridization). Furthermore, methylene blue (MB) is easy to synthesize, inexpensive, requires a low potential (0.1–0.4 V), and is less carcinogenic; therefore, it has gained our interest. In our previous work, our developed electrochemical DNA biosensor was able to distinguish significantly between the MB reduction current signals from non-complementary and complementary targets related to DNA sequences of dengue virus. However, most earlier MB research focused on synthetic DNA sequences or purified samples, and the use of MB for electrochemical detection based on large DNA sequences from real samples is currently still limited.

Most recent works related to electrochemical DNA biosensors have focused on studying miniaturized electrochemical sensor devices,^22^ amplification signals,^23,24^ biorecognition probes with new detection mechanisms,^25^ and the optimization of immobilization and hybridization conditions.^26^ However, far too little attention has been focused on the preparation of genomic DNA samples for electrochemical detection. This is due to constraints, as relying on the isolation and amplification of genomic genes *via* PCR-based laboratory procedures could restrict the application of electrochemical DNA biosensors in point-of-care testing. Much effort has been aimed towards obtaining miniaturized PCR-based detection devices for the fast, highly accurate, sensitive, and on-site detection of bacterial pathogens, viruses, parasites, and disease-causing agents. Several commercially available miniaturized PCR devices have been successfully utilized and documented in previous work. For example, miniPCR (www.minipcr.com), a portable PCR thermocycler, was successfully used by González-González *et al.*^27^ for the on-site amplification of SARS CoV-2 DNA sequences before detection using a commercial 96-well plate reader. Nguyen *et al.*^28^ reported using a handheld quantitative PCR machine from Biomeme Inc. (PA, USA) (www.biomeme.com), producing results comparable to traditional PCR-based laboratory assays for amplified gene sequences of *Flavobacterium psychrophio.* The successful use of the Biomeme PCR machine as portable PCR apparatus for the rapid testing of COVID-19 was also documented in the work of Zowawi *et al.*^29^ Other previous works have shown the successful design and development of portable PCR techniques, such as RT-PCR-assisted lab-on-a chip-based optical detection,^30^ portable rotary real-time fluorescent PCR,^31^ a miniaturized PCR-based portable bioaerosol,^32^ and others. These breakthroughs pave a path for the future development of miniaturized PCR-based electrochemical biosensors, which have the potential to revolutionize healthcare, particularly in dengue diagnostics. To the best of our knowledge, there has been little research focusing on genomic DNA sample preparation for electrochemical sensing applications, and this may be an interesting topic to investigate. In this paper, we demonstrate two strategies for the preparation of non-amplified and amplified genomic dengue gene samples and their application in detection based on an electrochemical DNA biosensor.

## 2. Experimental

### 2.1. Dengue virus culturing and isolation

**Fig. 1:**
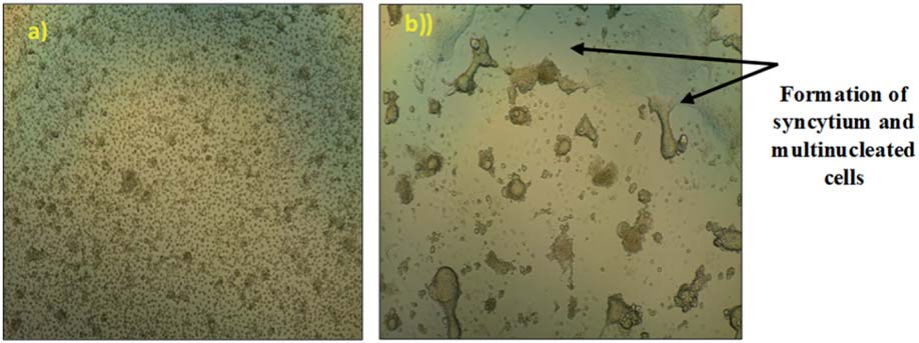
(a) An uninfected monolayer of C6/36 cells after achieving confluence (at 10× magnification). (b) A dengue-infected monolayer of C6/36 cells six days after inoculation with dengue virus stock solution (at 10× magnification).

Dengue virus type 2 (New Guinea C, (NGC) M29095) and an *Aedes albopictus* mosquito cloned cell line (C6/36) (CRL-1660, ATCC) were kindly provided by Dr Rafidah Hanim Syueb from the Department of Medical Microbiology and Parasitology, School of Medical, Universiti Sains Malaysia (USM), Malaysia. C6/36 cell lines were grown in Leibowitz L-15 medium (Life Technologies, France) supplemented with 5% fetal bovine serum (FBS) (PAA, Laboratories) at 28 °C for 96 h in a T75 flask (Sigma-Aldrich, USA). When the C6/36 cells reached confluence of 70–80%, the used medium was discarded, and the cells were rinsed with phosphate-buffered saline (PBS) (Sigma-Aldrich, USA), with the outcome shown in Fig. 1a. Then, the mosquito C6/36 cell lines were inoculated with 300 μL of dengue virus stock solution diluted in 3 mL of L-15 medium and incubated for 90 min at 28 °C. After that, the used medium was replaced with fresh L-15 medium with 1% FBS and propagated for six days. After six days, the uninfected C6/36 cell line (control) was compared with the infected C6/36 cell line. The infected C6/36 cell line showed the cytopathic effect (CPE) of dengue virus, and it can be characterized based on the formation of syncytia and multinucleated cells (Fig. 1b).

### 2.2. The extraction of dengue virus genomic RNA from cell cultures

**Scheme 1:**
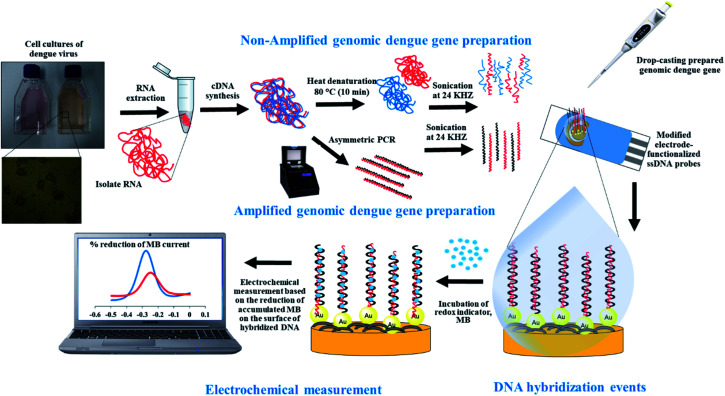
Non-amplified and amplified genomic dengue gene preparation for electrochemical sensing applications.

Genomic RNA was extracted from the dengue virus stock solution and dengue-virus-spiked human serum (ratio of culture stock to human serum, 1 : 5 (v/v)) using an Analytical innuPREP virus RNA extraction kit (Analytik Jena BioSolutions, Jena, Germany). Briefly, 150 μL of dengue virus stock solution was mixed vigorously with 450 μL of RL lysis solution in a 1.5 mL reaction tube for 10 s and incubated at room temperature (15 min). The lysed sample was mixed with 600 μL of RBS binding solution and vortexed for 10 s. A 650 μL lysed sample was pipetted out and added into a spin filter located in a 2.0 mL receiver tube. The cap of the spin filter was closed and centrifuged at 12 000 rpm for 1 min. The receiver tube was discarded, and the spin filter (containing the RNA sample) was put into a new 2.0 mL receiver tube. The cap of the spin filter was opened and 650 μL of HS washing solution was added, this was then centrifuged at 12 000 rpm for 1 min. The obtained dengue virus genomic RNA was further used for the subsequent analysis, as shown in Scheme 1.

### 2.3. The preparation of non-amplified dengue virus genomic RNA for electrochemical sensing

According to the manufacturer instructions, the extracted dengue virus genomic RNA was then reverse transcribed to cDNA using a Tetro cDNA synthesis kit (Bioline Pty Ltd, NSW, Australia). The components, including 10 μL of total RNA (up to 5 μg), 1 μL of specific primer dengue virus, 4 μL of 10 mM dNTP mix, 1 μL of 5× reverse transcribed buffer, 1 μL of Ribosafe RNase inhibitor, 2 μL of Tetro reverse transcriptase (200 U μL^−1^), and 2 μL of DEPC-treated water, were mixed gently by pipetting in a 100 μL RNase-free reaction tube. The mixture was incubated at 60 °C for 30 min. Then, the reaction was terminated *via* incubating the mixture at 85 °C for 5 min followed by rapid cooling in an ice bath. The quantity of cDNA was estimated as 17 ng μL^−1^*via* measuring OD260 using a UV-biophotometer (Eppendorf, Hamburg, Germany). The A260/280 OD ratio was between 1.8 and 2.2, verifying the quality of the cDNA samples. The cDNA samples were stored at −20 °C before directly detecting the non-amplified dengue virus genomic RNA using our developed biosensor.

### 2.4. The preparation of amplified genomic dengue virus gene samples using a reverse transcribed-polymerase chain reaction (RT-PCR) protocol and its optimization

The RT-PCR protocol was performed with a slight modification from the suggested MyTaq™ one-step RT-PCR kit (Bioline Pty Ltd. NSW, Australia) protocol. The components, including 25 μL of myTaq one-step mix, 2 μL of forward primer (GGC GYT CTG TGC CTG GAW TGA TG) (10 μM), 2 μL of reverse primer (AAG GAC TAG AGG TTA KAG GAG ACC C) (10 μM), 0.5 μL of reverse transcriptase, 1.0 μL of RiboSafe RNase inhibitor (10 U μL^−1^), 9.5 μL of DEPC water, and 10 μL of RNA extract, were mixed gently by pipetting in a 100 μL RNase-free reaction tube. The RT-PCR reaction was performed over 35 cycles using a PTC-200 Thermal Cycler machine (MJ Research Inc., Watertown, MA) with the following program: 1 cycle at 50 °C for 20 min (reverse transcription process), one cycle at 95 °C for 1 min (polymerase activation process), 35 cycles at 95 °C for 10 s (denaturation), 60 °C for 10 s (annealing), and 72 °C for 1 min (extension). The RT-PCR product (amplified cDNA) concentration was estimated as 420 ng μL^−1^ based on measuring OD_260_ using a UV-biophotometer (Eppendorf, Hamburg, Germany). The amplification of cDNA was analyzed *via* agarose gel electrophoresis. The gel bands were visualized using a UV transilluminator (Cole-Parmer, USA) and quantified using Image J software, version 1.49t (http://imagej.nih.gov/ij). For optimization studies, several parameters were studied, such as the annealing temperature of the RT-PCR protocol, the sonication time, and the reverse/forward (R/F) primer ratio concentration to optimize the RT-PCR products before they were used. Different annealing temperatures in the RT-PCR protocol (55 °C, 56 °C, 57 °C, 58 °C, 59 °C, 60 °C, 61 °C, 62 °C, 63 °C, 64 °C, 65 °C, 66 °C, 67 °C, 68 °C, and 69 °C) were used and examined *via* gel electrophoresis to determine the optimum conditions for amplified cDNA production. The final amplified cDNA samples were treated with different sonication times (5 s, 10 s, 25 s, 50 s, 100 s, and 200 s) for genomic DNA fragmentation. In addition, the optimization of asymmetric RT-PCR was performed as described above with slight modification, where the reverse primer concentration was adjusted to 0.1 μM, 0.2 μM, 0.4 μM, 1 μM, 2 μM, and 4 μM, while the forward primer concentration was fixed at 10 μM. The final asymmetric RT-PCR products were subjected to sonication (frequency: 24 kHz).

### 2.5. The detection of non-amplified and amplified dengue virus genomic samples using the developed DNA biosensor

The prepared non-amplified and amplified genomic dengue virus gene samples were treated before being further employed in our electrochemical DNA sensing set-up. The non-amplified genomic dengue virus gene samples were denatured into single-stranded DNA form *via* heating to 80 °C for 10 min followed by rapid cooling at 4 °C for 10 min. The non-amplified genomic dengue virus gene and amplified genomic dengue virus gene samples were subjected to sonication (24 kHz frequency) for 5 min. The treated non-amplified and amplified genomic dengue samples were then ready to be employed in our developed biosensor system.

The fabrication and mechanism of our developed DNA biosensor have been described in our previous work.^11^ Firstly, a gold working electrode (GWE) based on a disposable screen-printed gold electrode (SPGE) (DropSens, Spain) was polished carefully using 3 μm alumina powder and subsequently cleaned with distilled water and dried with nitrogen gas (N_2_). The GWE surface was then treated with a solution made up of H_2_O, NH_4_OH (30%), and H_2_O_2_ (30%) (5 : 1 : 1) for two minutes before being functionalized with silicon nanowires (SiNWs). 6 μL of silicon nanowire (SiNW) suspension (1 × 10^6^ wires per mL) in 0.5% APTES was cast onto the GWE surface and this was incubated for three hours at room temperature before being washed with ethyl-ethanol and cured at 100 °C for 30 min. The SiNW-functionalized SPGE was decorated with prepared gold nanoparticles as described in previous work. This fabricated electrode was drop-cast with 10 μL of a thiolated single-stranded DNA (ssDNA) probe (5′ SH-(CH_2_)_6_-AAC AGC ATA TTG ACG CTG GGA GAG ACC-3′) at a concentration of 3 μM and incubated for 24 h at room temperature. This was followed by washing unbound thiolated DNA with Tris–EDTA (TE) (20 mM Tris–HCl, 1 mM EDTA) three times. For DNA hybridization events, 10 μL of treated genomic dengue virus gene solution (non-amplified or amplified) was drop-cast onto the fabricated electrode surface, allowing for DNA hybridization events with the immobilized DNA probe on the fabricated electrode surface.

These DNA hybridization events were measured *via* soaking the fabricated electrode in 50 mM Tris–HCl at pH 7.6 containing 50 μM MB without applying any potential for 20 min at room temperature, followed by washing with Tris–HCl buffer to remove any excess unbound MB, and drying with N_2_ gas. Then, the fabricated electrode was connected to a potentiostat to carry out differential pulse voltammetry (DPV) in blank buffer (50 mM Tris–HCl at pH 7.6) in the potential range of −0.5 V-0 V, with a potential step-size of 0.005 V, a modulation amplitude of 0.5 V, and an interval time of 0.64 s, at room temperature. The change in the MB reduction current before and after the introduction of the genomic dengue virus gene sample was used for measuring the hybridization efficiency. The hybridization efficiency is calculated as follows:1
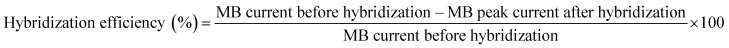


## 3. Results and discussion

### 3.1. The direct detection of non-amplified genomic dengue virus gene samples from cell cultures

It is worth emphasizing that there are specific issues related to the direct detection of non-amplified genomic dengue virus gene-based hybridization events *via* a DNA biosensor approach. In this case, we highlight the main issues. Firstly, the complexity of the genomic dengue virus gene, consisting of ∼11 000 base pairs (bps) as reported by Lindenbach and Rice,^33^ creates challenges when samples have to hybridize with a specific biorecognition or immobilized 27-mers-DNA probe on the fabricated electrode. This is because the higher the molecular weight size of a genomic or nucleic acid sample, the higher the levels of steric hindrance and electrostatic repulsion between negative phosphate groups, which can reduce and hinder hybridization events on the fabricated electrode. In contrast to the DNA structure, the genomic dengue virus gene is in single-stranded RNA (ssRNA) form, which is not a stable state due to the lack of sugar hydroxyl groups, making it highly susceptible to alkaline hydrolysis and chemical and cellular RNase degradation.^34^ This issue is frequently a major concern when hybridizing with specific immobilized DNA probes in DNA biosensor systems due to instability when exposed to harsh environments, whether the genomic RNA is in good condition or not before being applied. This can lead to inconsistent results and false positives. Furthermore, the low amount of genomic nucleic acid material present in real samples is difficult to detect using DNA biosensors, which also becomes a major barrier to real samples detection.^35^

In this work, we anticipate overcoming all these mentioned issues *via* focusing on sample treatment steps, such as cDNA conversion, heat denaturation, and sonication steps, before further employing our developed biosensor system. Due to RNA stability issues, the ssRNA genomic dengue virus gene sample is converted to complementary DNA (cDNA) using a reverse transcriptase enzyme. The dengue virus cDNA was subjected to sonication treatment for 5 min and heat-denaturing treatment (boiling at 80 °C for 10 min followed by rapid cooling in an ice bath for 1 minute) to ensure that double-stranded dengue virus genome material in cDNA is denatured before allowing it to hybridize with the DNA probe for hybridization detection. Our findings demonstrate that our developed DNA biosensor (*n* = 10) generated a higher MB current after the introduction of treated genomic dengue virus gene samples (17 ng μL^−1^), where it increased from 0.92 μA to 1.20 ± 0.09 μA (RSD: 6.94%, *n* = 10) (Table 1).

**Table 1:** The MB currents from the developed DNA biosensor upon the direct detection of non-amplified genomic dengue virus gene samples

MB current signals from the developed DNA biosensor	
MB current signal in the absence of non-amplified genomic dengue virus (μA)	MB current signals after the introduction of non-amplified genomic dengue virus (μA)
0.92 ± 0.034	1.22
	1.25
	1.28
	1.23
	1.15
	1.13
	1.10
	1.29
	1.32
	1.04
	Average (*n* = 10) **1.20 ± 0.09**

In this study, we noticed that the MB current behaviour based on our electrochemical sensor differs from our previous work, in which a lower signal was generated after introducing dengue virus oligonucleotide DNA (hybridization events).^10^ These differences can be explained due to the different binding affinities of MB toward short oligonucleotides and genomic dengue virus gene bases. It is known that MB bound to dsDNA (hybridized) results in a decreased MB current compared to ssDNA (before hybridization) due to the inaccessibility of guanine bases to MB.^36–41^ However, these previous studies demonstrated fabricated DNA sensors for the hybridization detection of short DNA sequences or oligonucleotide targets (less than 30 bps) with the same length as the immobilized DNA probes.

In this study, our developed DNA biosensor was employed to detect genomic dengue virus gene samples in which the length of the genomic sequence (∼11 000 bps) is much longer than our immobilized DNA probe sequence (∼27 bps). The hybridization events between the immobilized DNA probe and the longer genomic dengue virus gene would expose more guanine bases (unhybridized bases) in the target genomic sequence. As a result, MB would bind more to unhybridized guanine bases, leading to high MB accumulation that needs to be reduced, producing a high MB current signal after hybridization. Furthermore, the increased accumulation of MB is also driven by a greater number of negatively charged phosphate groups in the larger genomic dengue virus sample, which may increase electrostatic attraction toward positively charged MB.^42^ Thus, an increase in the MB current signal after incubation with longer genomic dengue virus gene sequences is anticipated. Our findings were comparable with the previous work of Solanki *et al.*,^43^ who successfully fabricated a DNA electrochemical sensor based on nanostructured zirconium oxide (NanoZrO_2_) modified ITO to detect genomic *Escherichia coli* using MB as a redox indicator. The authors noticed a significant reduction in the MB current when incubating with a short complementary *E. coli* oligonucleotide sequence.

Interestingly, the MB current increased tremendously after hybridization between genomic *E. coli* and ssDNA/NanoZrO_2_/ITO. Singh *et al.*^44^ reported that the MB current was found to be higher in the presence of fragmented genomic DNA *Salmonella typhi* from a blood sample when utilizing a gold nanoparticle aggregate/ITO electrode. In contrast, some previous works also exhibited MB current reduction after incubating an immobilized ssDNA-modified electrode with genomic DNA sequences from real samples.^44–47^ This could be explained based on the fact that their target genomic sequences were first fragmented into short genomic DNA sequences *via* a sonication approach. Although our findings and other previous work demonstrate that the use of a fabricated DNA biosensor can directly recognize non-amplified genomic sequences without the need for a PCR approach, there are some issues. Due to the larger genomic dengue virus gene introduced in our developed DNA sensing approach, false positive results are possible due to non-specific sequence hybridization between our immobilized DNA probe and non-specific fragments of genomic dengue virus. As a result, it is unclear whether the increase in the MB redox current results from specific or non-specific hybridization, unless the specific sequences of interest to be hybridized with DNA probe sequences are successfully identified.

### 3.2. The amplification of genomic dengue virus gene samples using a reverse transcriptase polymerase chain reaction (RT-PCR) technique for DNA electrochemical detection

The coupling of the reverse transcriptase-polymerase chain reaction (RT-PCR) technique with our developed DNA sensor was employed in this work. Using the RT-PCR amplification approach for amplified genomic sample preparation before detection with our developed DNA biosensor could overcome the previous issues. Several parameters were investigated, including the RT-PCR annealing temperature, amplified genomic dengue virus gene sonication time, and use of asymmetric PCR, to enhance the detection of genomic dengue virus.

### 3.3. The effect of annealing temperature during RT-PCR amplification

**Fig. 2:**
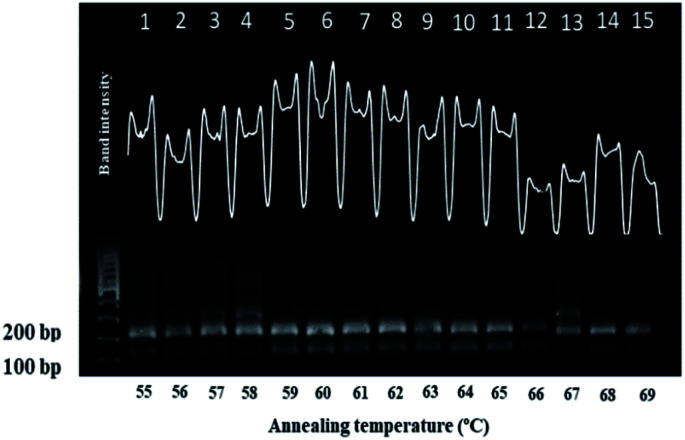
The effects of various annealing temperatures (T4) on the RT-PCR-based amplified genomic dengue virus gene approach.

The success of RT-PCR amplification relies on the specificity of the primer toward its specific target gene when annealing. According to Hwang *et al.*,^48^ varying the annealing temperature (*T*_a_) can influence the specificity of the annealing primer *via* altering the base pairing between the primer and specific gene regions. Therefore, *T*_a_ was optimized to achieve high efficiency and specificity in the amplified genomic dengue virus gene. This is because below the optimum *T*_a_ value, non-specific primer binding may occur, thus leading to unwanted or non-specific genomic gene amplification.^49–51^ Meanwhile, if *T*_a_ is too high, this could lead to low yield of genomic gene amplification products due to the low efficiency of annealing primer to the specific DNA gene region target.^52–55^ The optimization of *T*_a_ for dengue virus amplification using gradient PCR was performed with a range of *T*_a_ values from 55 °C to 69 °C. As shown *via* gel electrophoresis analysis, all tested *T*_a_ values give amplification of the products of interest relating to dengue virus (115 kb and 195 kb) (Fig. 2). However, the amplification of genomic dengue was poor at a *T*_a_ value between 55 °C and 58 °C (lanes 1–4) due to the appearance of multiple bands resulting from non-specific genomic amplification. Image J software was employed to show the band intensities for each *T*_a_ value to observe gel electrophoresis clearly (inset of Fig. 2). It was shown that high-efficiency genomic dengue virus gene amplification was obtained at a *T*_a_ value of 60 °C (lane 6), which give the highest band intensities from gel electrophoresis. Further increasing *T*_a_ above 65 °C also reduced the yield of PCR products, where the intensities of bands were dramatically decreased from 60 °C to 65° due to poor primer annealing. Some previous researchers have argued that *T*_a_ is not a critical factor in genomic gene amplification since no significant changes were observed in their desired band intensities.^55–57^ Due to obvious changes of band intensities generated here, it could be considered that *T*_a_ plays an important role in genomic dengue gene amplification for our developed DNA sensor application. Hence, a *T*_a_ value of 60 °C was identified as optimal for genomic dengue gene amplification in subsequent experiments.

### 3.4. The effects of sonication time on amplified genomic dengue virus gene samples

**Fig. 3:**
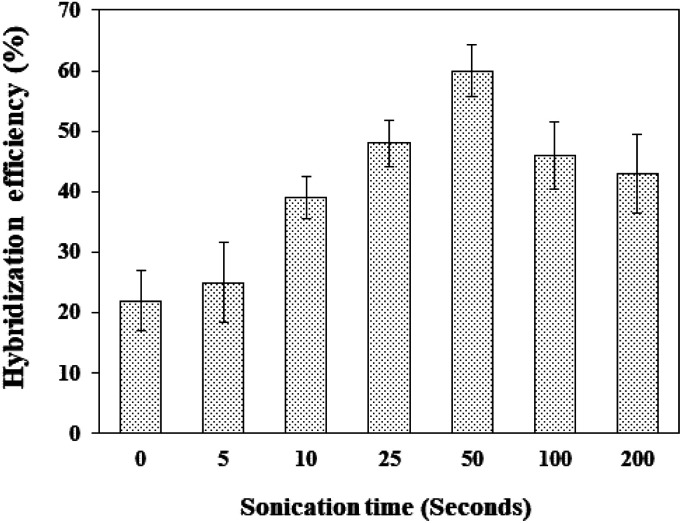
The effects of sonication time on the hybridization efficiencies of amplified genomic dengue virus gene samples.

Genomic fragmentation treatment *via* a sonication method was employed to improve the accessibility of the amplified genomic dengue sample *via* generating short genomic fragments to hybridize with our immobilized DNA probe on the fabricated electrode surface. This sonication treatment has been applied in genomic gene detection using various types of DNA biosensors, such as in optical,^58,59^ acoustic,^60^ surface plasmon resonance (SPR),^61^ electrochemical,^46^ and electrical^62^ approaches. The generation of an acoustic shear force for genomic DNA fragmentation is widely used due to the ease of operation and rapidity.^63–66^ The influence of sonication time on amplified genomic dengue virus gene (420 ng μL^−1^) preparation, ranging from 5 s to 200 s (24 kHz frequency), is shown in Fig. 3.

The hybridization efficiency was improved upon increasing the sonication time of amplified genomic dengue virus gene samples from 5 s to 50 s. This could probably be attributed to the sonication approach successfully breaking the amplified genomic dengue virus gene samples into smaller fragments, resulting in an improved DNA hybridization efficiency signals being obtained with our developed DNA sensor. This finding is in agreement with Liu *et al.*,^62^ who showed that the gel electrophoresis patterns of genomic DNA fragments were reduced in size from 2000 bp to 200 bp after 5 minutes of sonication treatment. Their group noticed an improvement in the DNA hybridization signal after sonication treatment in the case of *Bacillus cereus* gDNA detection. Generally, a shorter amplified genomic dengue virus gene length was generated when a longer sonication time was used to treat an amplified genomic dengue virus gene sample.

In contrast, amplified dengue virus detection without sonication treatment exhibited a low DNA hybridization efficiency of 25%. Fig. 3 depicts that 50 s was the optimum sonication time, achieving an optimum hybridization efficiency of 57% (RSD: 8.65%) and above 50 s of sonication, the hybridization efficiency begins to decrease. This result revealed that sonication treatment plays an important role in sample preparation for DNA electrochemical sensing, enhancing DNA hybridization and thus improving the current signal during real sample detection.

### 3.5. The effects of the reverse to forward (R/F) primer concentration ratio in asymmetric PCR for the amplification of single-stranded DNA (ssDNA) amplified genomic dengue virus gene samples

Previous research has shown that our developed DNA sensor coupled with RT-PCR amplification can successfully detect genomic dengue virus from cell cultures. However, the RT-PCR-amplified genomic dengue virus gene products are usually in double-stranded DNA (dsDNA) form, requiring additional steps to separate dsDNA (heat-denaturing) into ssDNA sequences before allowing hybridization to occur. It is possible for this separated ssDNA to re-anneal with each other instead of hybridizing with the immobilized DNA probe, resulting in a decrease in sensitivity and poor reproducibility.^67,68^ Modified RT-PCR amplification, known as asymmetric PCR, coupled with a DNA sensor has been extensively used due to its higher sensitivity, as it generates an excess amount of the ssDNA target for direct hybridization with an immobilized-DNA-probe-modified electrode.^69–74^ The reverse-to-forward (R/F) concentration ratio is an important part of the asymmetric PCR protocol for creating excess ssDNA production.^75^ This is because in asymmetric PCR, the primer with a lower concentration is involved in the production of dsDNA, whereas the primer with a higher concentration (which does not bind to any template) is responsible for the production of ssDNA.^76^

**Fig. 4:**
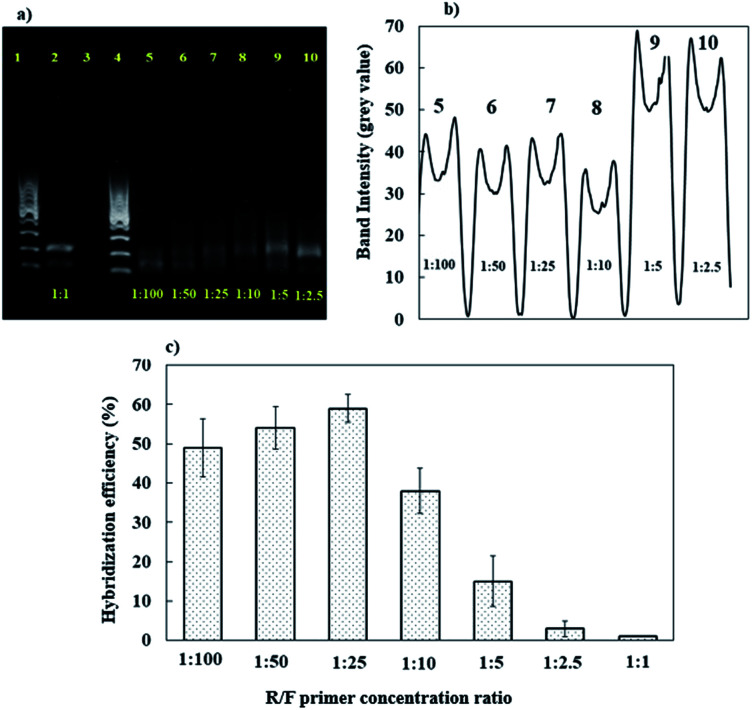
(a) 1% agarose gel electrophoresis analysis at different R/F primer concentration ratios for the amplification of the PCR product. Lane 1: 1000 base pair ladder; lane 2: conventional PCR (1 : 1); lane 3: negative control; lane 4: 1000 base pair ladder; lane 5: 1 : 100; lane 6: 1 : 50; lane 7: 1 : 25; lane 8: 1 : 10; lane 9: 1 : 5; lane 10: 1 : 2.5. (b) The band intensities of the asymmetric RT-PCR products with varying R/F primer concentration ratios. (c) The effects of the R/F primer concentration ratio on the hybridization efficiency of the developed sensor.

The conventional RT-PCR amplification protocol applied used same amounts of reverse and forward primer (1 : 1) for obtaining the amplified PCR product in dsDNA form. Hence, the optimization of asymmetric PCR was carried out *via* adjusting the concentration of reverse primer from 0.1 to 4 μM while forward primer concentration was fixed at 10 μM. Fig. 4a and b show gel electrophoresis analysis of the conventional PCR product (lane 2) and asymmetric PCR products (lanes 5–10) from dengue virus gene samples. The band intensities of the conventional PCR product (lane 1) are higher and thicker than the asymmetric PCR products (lanes 5–10) (Fig. 4b). This is due to the large amount of dsDNA generated by conventional PCR compared with asymmetric PCR.^74^ Similarly, as reported in ref. 77, it has been observed that the band intensities of asymmetric PCR products can be reduced by half in comparison with conventional PCR due to excess ssDNA instead of dsDNA production.

For ssDNA production *via* an asymmetric PCR approach, the band intensities became stronger with an increase in the reverse primer concentration when the forward primer concentration was fixed (Fig. 4a and b). The R/F concentration ratios of 1 : 2.5 (lane 10) and 1 : 5 (lane 9) exhibit high band intensities from asymmetric PCR products. However, this finding does not reflect the optimal production of a ssDNA dengue gene sample for DNA sensor applications. This can probably be attributed to asymmetric PCR products containing different proportions of ssDNA and dsDNA, as reported by Avci-Adali *et al.*,^78^ which could probably affect the band intensities during gel electrophoresis. Therefore, the best R/F primer ratio concentration for optimal amplification during ssDNA dengue gene production was evaluated based on the hybridization efficiency using our developed DNA sensor. Fig. 4c demonstrates the effects of the R/F primer concentration ratio on the DNA hybridization efficiency. In this work, all the asymmetric PCR ssDNA dengue gene products were sonicated first and direct used for electrochemical detection using our developed DNA sensor without a heat-denaturing treatment step. It is assumed that if a high amount of amplified ssDNA dengue gene target is available, this would improve the DNA hybridization efficiency signal. Thus, this could reflect the effectiveness of the asymmetric PCR approach for preparing an excess amount of ssDNA dengue gene target for DNA sensor applications.

In contrast to gel electrophoresis, the R/F primer concentration ratios of 1 : 5 and 1 : 2.5 resulted in low DNA hybridization efficiencies, suggesting that the ssDNA production yield was lower at these ratios. Meanwhile, the direct detection of conventional PCR products using an R/F primer concentration of 1 : 1 results in no signal from DNA hybridization due to the high background of the MB signal. This observation is because the conventional PCR product was in the form of a dsDNA dengue gene sample, which is inaccessible for interactions with the DNA probe, requiring heat-denaturing treatment before DNA hybridization detection. Hence, it was concluded that a high R/F ratio of 1 : 25 was enough to amplify the ssDNA dengue gene target using the asymmetric PCR approach for the direct electrochemical detection of the hybridization reaction using our developed DNA sensor. With this asymmetric PCR approach, we could directly prepare genomic ssDNA dengue gene samples in a single reaction without a denaturation step, minimizing the time and operation cost.

### 3.6. The analytical performance of the developed sensor during real sample detection

**Fig. 5:**
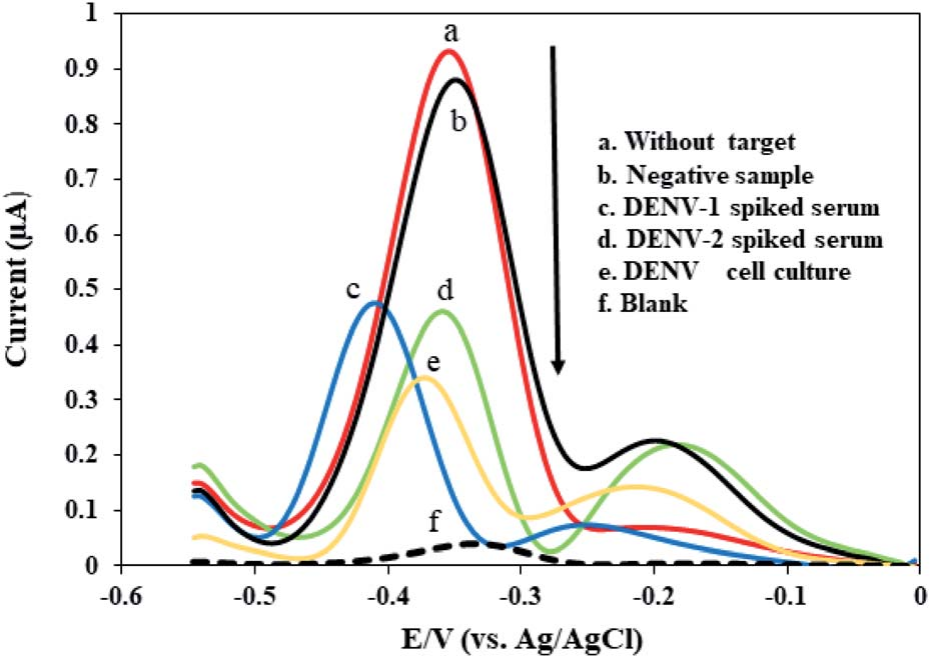
The MB current responses of the developed DNA biosensor coupled with the optimized asymmetric PCR approach for the detection of different kinds of genomic dengue virus gene samples.

After the successful preparation of amplified ssDNA dengue virus samples using an asymmetric PCR protocol, the immobilized-DNA-probe-functionalized fabricated electrode was hybridized with different kinds of genomic DNA from a negative sample, a serum sample spiked with dengue virus type 1, a serum sample spiked with dengue virus type 2, and a dengue virus culture isolate sample, as presented in Fig. 5. The DPV response of a bare modified electrode displayed a small peak current, thus revealing the absence of MB accumulation on the electrode surface (curve f in Fig. 5). However, after the DNA probe was immobilized on the surface of the fabricated electrode, the MB redox current was enhanced (curve a in Fig. 5). This result proved that MB has high affinity for the DNA sequence on the surface, resulting in a high MB redox current. The immobilized-DNA-probe-functionalized fabricated electrode does not show a noticeable current change after treatment with a negative sample. This negative sample contains human serum that is free from dengue virus. As a result, the asymmetric PCR product does not contain specific complementary dengue virus gene material that can hybridize with our DNA probe. Thus, a high MB redox current close to the DNA probe current is anticipated, indicating that no hybridization occurred (curve b in Fig. 5). Similar observations during the detection of amplified PCR products have been reported in the previous literature.^79–81^

Meanwhile, a significant decrease in the MB redox current is obtained upon incubating with amplified genomic ssDNA targets from human serum samples spiked with dengue virus type 1 and 2, and a dengue virus cell culture sample (curves c, d, e, respectively, in Fig. 5). The MB redox current was reduced during hybridization due to a lack of MB bound to the guanine bases, as described above. It was observed that our developed DNA sensor did not discriminate between the MB redox currents obtained from amplified ssDNA samples of dengue virus types 1 and 2, showing good potential for detecting these serotypes. It is expected that the MB redox current of the dengue virus cell culture sample (curve e, Fig. 5) is lower than the currents from the human serum samples spiked with dengue virus (curves c and d, Fig. 5) most probably because the dengue virus cell culture could provide a high amount of the ssDNA target. In general, these findings demonstrate that our developed DNA sensor shows good selectivity and is sufficient for detecting dengue virus, being capable of discriminating between the MB redox current signals of negative and positive samples.

## 4. Conclusions

Further work on utilizing newly synthesized redox indicators for the electrochemical detection of DNA hybridization events is required. A new redox indicator based on intercalation binding is suggested due to it being highly specific to dsDNA (after hybridization) through intercalation between G-C or A-T base pairs. A novel redox indicator of this type could overcome the limitations of MB seen in this work, as MB is affected by the length and base composition of the DNA sequences. Due to rapid advancements in the field of nanotechnology, new electroactive-indicator-functionalized nanomaterials can be further explored to improve the electrochemical signals from low-concentration samples and specificity towards analytes. In this study, we successfully demonstrated and highlighted the critical parameters relating to an RT-PCR approach for the preparation of amplified genomic dengue virus gene samples coupled with our developed electrochemical DNA sensor.

Despite all the process steps and materials used, the system is good and stands out in this area. Despite the fact that a genomic preparation technique based on PCR is still required to use our developed sensor, in this study we were able to directly prepare genomic ssDNA dengue virus gene samples in a single reaction without a denaturation step, which is an improvement over traditional PCR methods. The optimized genomic dengue virus gene preparation approach based on asymmetric PCR, when coupled with our DNA biosensor, can successfully distinguish between the current signals obtained from different kinds of genomic dengue virus gene samples. The combination of this sample preparation approach with our developed electrochemical biosensor offers an alternative system to traditional methods based on gel electrophoresis visualization techniques and ELISA assays that are used in practical settings (hospitals, laboratories, *etc.*), showing greater specificity and sensitivity, a rapid response, and ease of operation. Thanks to the burgeoning miniaturized PCR technology field, miniaturized PCR-based biosensors for the fast diagnosis of dengue virus could be realized in the future.

## Conflicts of interest

No conflicts of interest are declared.

## Supplementary Material
